# Clinical applications of chlorhexidine in oral wound healing: Scoping review of current evidence and research gaps

**DOI:** 10.4317/medoral.27864

**Published:** 2025-11-22

**Authors:** Elena Bucataru, María de Nuria Romero-Olid, Pablo Ramos-García, Miguel Ángel González-Moles

**Affiliations:** 1 School of Dentistry, University of Granada, Granada, Spain

## Abstract

**Background:**

This scoping review aimed to comprehensively synthesize the current evidence and gaps regarding the use of chlorhexidine (CHX) in oral wound healing after surgical procedures.

**Material and Methods:**

PRISMA-ScR reporting guidelines were followed. A systematic search of MEDLINE, Embase, Web of Science, and Scopus was conducted to identify primary-level studies published before July-2024.

**Results:**

Sixty-six studies, encompassing 6,763 patients met eligibility criteria. Third molar extractions were the most studied procedures (n=44), where CHX use consistently improved wound healing, significantly reduced the risk of alveolar osteitis and alleviated postoperative pain, with over 70% of studies reporting favorable outcomes. CHX application, across different concentrations and pharmaceutical forms, was consistently associated with better wound healing, with gels (n=29) and rinses (n=36) showing favorable results in 75% and 80% of studies, respectively; both 0.12% (n=21, 72.7%) and 0.20% (n=41, 81.3%) concentrations proved effective, with short-term postoperative regimens (7 days, n=27) or intraoperative application (n=22) emerging as the most reliable protocols. Moreover, combinations of CHX with adjuvant agents, particularly chitosan (n=4) and hyaluronic acid (n=5), demonstrated synergistic benefits, although the number of available trials remains limited.

**Conclusions:**

In conclusion, current evidence supports the application of CHX as an effective and low-cost adjuvant in optimizing oral wound healing and preventing postoperative complications, singularly alveolar osteítis and postoperative pain after third molar extractions. Both gel and rinse formulations, as well as concentrations of 0.12% and 0.20%, have been shown to be effective, with indications related to clinical context and duration of use. Finally, CHX associatiations with chitosan and hialuronic are promising, although still based on a low evidence level. Future well-designed clinical trials are needed to address current evidence gaps, standardize administration protocols and further explore synergistic combinations.

## Introduction

The repair of surgical wounds in the oral cavity involves a multifaceted and dynamic biological process designed to restore both structure and function to the injured tissues (1 , 2). This process involves four sequential and overlapping phases: Hemostasis, inflammation, proliferation, and tissue remodeling. During these stages, several cellular events occur, including vasoconstriction, vasodilation, neoangiogenesis, migration and proliferation of fibroblasts, epithelial and endothelial cells, formation of granulation tissue, and extracellular matrix deposition (1 , 3 - 5). While this repair mechanism is finely regulated under physiological conditions, it may be significantly impaired by systemic disorders (e.g., diabetes mellitus or hypothyroidism (1 , 6)), insufficient vascularization, presence of necrotic tissue or infectious processes. In particular, surgical wounds within the oral cavity are highly susceptible to bacterial contamination due to the rich and diverse oral microbiota, increasing the risk of healing complications such as delayed epithelialization, dehiscence, pain, or alveolar osteítis (7). One of the most prevalent postoperative complications following oral surgery is alveolar osteitis, also known as dry socket, especially after third molar extraction. This condition, which can affect up to 30% of these procedures depending on the diagnostic criteria and patient-related risk factors (8), is characterized by severe pain, partial or total disintegration of the blood clot, and exposure of the alveolar bone, often accompanied by halitosis (9). The pathogenesis of alveolar osteitis involves both fibrinolysis and bacterial infection, with additional contributory factors including smoking, poor oral hygiene, traumatic tooth extraction, hormonal influence, use of vasoconstrictors, or due to inadequate surgical technique (8 , 9).

In an attempt to reduce the incidence of such complications and promote optimal wound healing, some antiseptic agents have been explored for topical application in surgical oral sites (10). Among them, chlorhexidine (CHX) has gained special relevance due to its broad-spectrum antimicrobial properties, rapid bactericidal effect, prolonged bacteriostatic activity, and relatively low toxicity (11). CHX acts by disrupting microbial cell membranes, leading to precipitation of cytoplasmic components and cell lysis (11). It also shows antifungal and partial virucidal activity (11). Its sustained action and high bioavailability have led to its widespread use in multiple pharmaceutical forms -mouthwashes, gels, sprays, toothpastes, and even bioadhesive chips- with the most common concentrations ranging between 0.12% and 0.20% (11 , 12). In clinical practice, CHX has been extensively used across oral surgical procedures, such as periodontal interventions, soft tissue biopsies, implant placement, and tooth extractions (12 , 13). Its application has been proposed to minimize inflammatory symptoms, pain, and the incidence of alveolar osteitis, thereby improving patients' postoperative quality of life (13). However, despite its extended clinical use and well-documented antimicrobial benefits, there is still no international consensus regarding the optimal CHX administration protocol in terms of dosage, pharmaceutical form, timing (pre- or postoperative), and duration of use (13). Moreover, the risk of side effects -such as taste disturbances, mucosal irritation, tooth staining, and even calculus formation- requires careful evaluation of its risk-benefit ratio (14 , 15). For these reasons, a better understanding of CHX effectiveness and safety profile in the context of oral wound healing is essential for evidence-based clinical recommendations.

Based on this background, the present scoping review aims to comprehensively explore the current clinical evidence derived from primary-level studies regarding the use of chlorhexidine in wound healing after oral surgery, focusing on relevant clinical outcomes (such as epithelialization, erythema reduction, wound dehiscence, postoperative pain, and the occurrence of alveolar osteitis), standardized administration protocols (CHX form, concentration and timing of application) and potential synergic combination with other agents. By synthesizing and organizing available knowledge, this investigation also seeks to identify evidence gaps, clarify inconsistencies in the literature, and offer updated recommendations for future research.

## Material and Methods

This scoping review adhered to the Preferred Reporting Items for Systematic reviews and Meta-Analyses extension for Scoping Reviews (PRISMA-ScR) (16). A study protocol was registered on Open Science Framework (OSF) and is publicly accessible (registration DOI: 10.17605/OSF.IO/ZKJ6E; link: https://osf.io/tkd5q). OSF is an open source platform where researchers can share protocols, data and contributes to transparency of research.

Search strategy

The databases MEDLINE (via PubMed), Embase, Web of Science and Scopus were searched for primary-level studies published before July-2024, with no lower date limit. The search strategy (Supplement 1. http://www.medicina.oral.com/carpeta/suppl1_27864) was designed and conducted by combining thesaurus terms used by the databases (i.e., MeSH and EMTREE) with free terms, designed and built to maximize sensitivity. An additional final screening was performed by handsearching the reference lists of retrieved included studies and using Google Scholar. All references were managed using Mendeley v.1.19.8 (Elsevier, Amsterdam, The Netherlands); and duplicate references were eliminated.

Eligibility criteria

We included randomized clinical trials (RCTs) and quasi-RCTs (q-RCTs), with parallel groups or split-mouth design, evaluating the application of CHX -alone or in combination with other antiseptics or antibiotics- for the experimental intervention, and placebo or absence of treatment for the control group regarding the risk of complications in wound healing after oral surgery procedures. If two research arms applying CHX at different concentrations or with different vehicles were investigated, both were included and considered as separate units of analysis. No restrictions were applied in relation to the publication language, publication date or population characteristics such as geographic area, age or gender.

The following exclusion criteria were applied: Retracted articles, non-randomized clinical trials or observational studies, case reports, preclinical experiments (animal experimentation or in vitro research), articles without scientific method and/or results (letters, editorials, personal opinions, commentaries, meeting abstracts, literature narrative reviews, or book chapters), as well as secondary/tertiary-evidence level studies (scoping reviews, systematic reviews with or without meta-analysis, overviews of reviews, umbrella studies, etc.); surgical procedures from anatomic areas distinct to the oral cavity; primary-level studies without control groups, or controls exposed to an experimental intervention with known antiseptic effect (e.g., CHX, antibiotics or other drugs); no analysis of clinical outcomes of interest or a lack of essential data for the statistical estimation of effect size metrics with their corresponding confidence intervals; inter-study overlapping populations.

Eligibility criteria were independently applied by two blinded reviewers (EB and MNRO). Any discrepancies were resolved by consensus with a third supervising author (PRG). Records were selected in two phases: First, titles and abstracts of articles that appeared to meet the inclusion criteria were screened; then, the full texts of the selected articles were reviewed to exclude those that did not meet the eligibility criteria.

Data extraction

Two authors (EB and MNRO) independently extracted data following full-text review and completed a standardized data collection form using Excel v.16 (Microsoft, Redmond, WA). The extracted datasets were subsequently compared, and discrepancies were resolved by consensus. Extracted data included: First author of the study, language and publication date, country, sample size, study design, type of oral surgery, CHX/AH concentration and vehicle, control group intervention, recruitment and follow-up periods, intervals between follow-up visits, as well as participants' gender and age, and outcomes of interest.

Critical analysis and evidence synthesis

The present scoping review methodology appears pertinent for the purpose of investigating for evidence-based findings and identifying potential gaps across areas where evidence may be lacking (17 , 18). In this sense, the clinical implications of chlorhexidine use in oral surgical wound healing and complications were explored, particularly: Application protocols, exploring which concentration (0.20%, 0.12%, etc.,), vehicle (gel or rinse) and application period or timing is the most appropriate choice; CHX effectiveness on additional outcomes such as alveolar osteitis, pain, wound dehiscence, healing progression, swelling, trismus, inflammation, edema, among others; and, due to the wide variability, all the wound healing indices used and its parameters. All these topics were investigated across primary-level studies, in order to synthesize current evidence, search for potential evidence gaps, and guide future research. Key results were shown in descriptive tables, using a systematic methodological approach, and discussed in depth.

## Results

Results of the literature search

**Figure 1:**
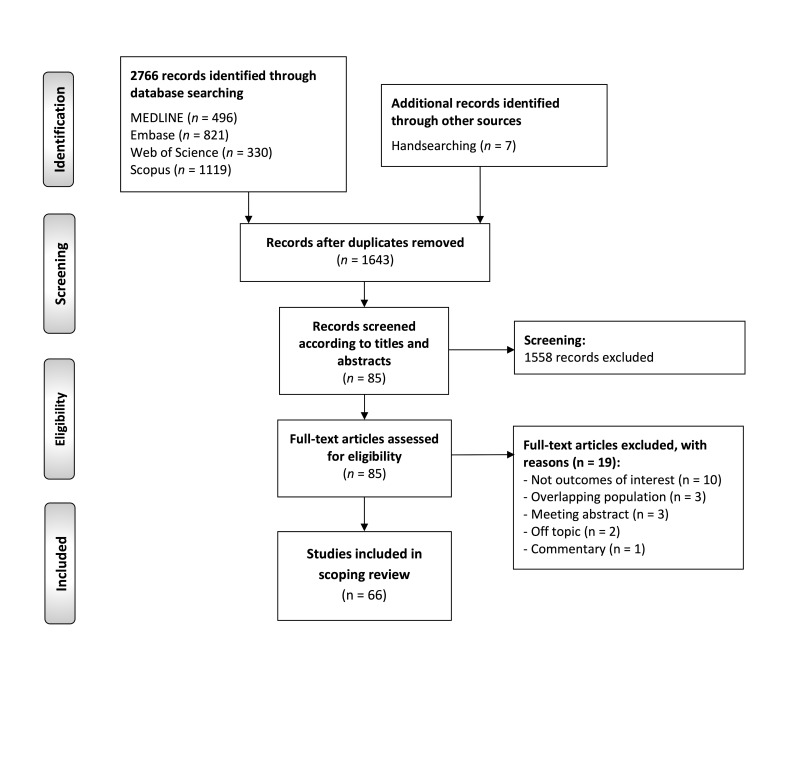
Flow diagram depicting the process of screening, identification and selection of primary-level studies included in this scoping review.

The flow diagram (Figure 1) illustrates the results of the literature search, study identification and selection process in this scoping review.

1

A total of 2766 publications were retrieved: 496 from MEDLINE (through PubMed), 821 from Embase, 330 from Web of Science, 1119 from Scopus and 7 through manual searching. After removing duplicates, 1643 records were considered potentially eligible and screened based on titles and abstracts, leaving a sample of 85 studies for full-text evaluation. Finally, 66 studies that met all eligibility criteria were included for critical analysis and evidence synthesis in our scoping review (the references of excluded records -with exclusion reasons- and the included studies can be found, respectively, in the Supplement 2 (http://www.medicina.oral.com/carpeta/suppl2_27864) and Supplement 3 (http://www.medicina.oral.com/carpeta/suppl3_27864).

Study characteristics

Table 1 provides an overview of the characteristics of the 66 studies included in this scoping review.

Table 1

The earliest study was published in 1979, and the most recent in 2024. All the studies are clinical trials -44 randomized and 22 quasi-randomized- focusing on patients (n=6763 cases) undergoing oral surgical procedures such as simple dental extraction, third molar surgery (impacted or not), periodontal surgery, oral incisions, biopsy and single implant placement with flap elevation, with or without relieving incision.

Although the oral surgery procedure studied varied among the studies, the most frequently explored procedure was third molar extraction -impacted or not- (n=44, 61.11%), followed by periodontal surgery (n=10, 13.88%), simple extraction (n=8, 11.11%), implant surgery (n=5, 6.9%), biopsy (n=3, 4.17%), and the least studied procedures were maxillofacial surgery (n=2, 2.7%).

Regarding the CHX intervention studied, the most investigated was the application of CHX alone (n=55). This was followed by studies evaluating CHX in combination with other products (n=10), CHX combined with antibiotics (n=5), CHX combined with hyaluronic acid (n=5), and, lastly, CHX combined with chitosan (n=4). Of the 66 articles, 36 assessed CHX in rinse form, 29 in gel form, and one did not report the vehicle used. Various concentrations of CHX were included, with the most frequently studied being 0.20% (n=41; 62.12%) and 0.12% (n=21; 31.81%). Both period and timing of CHX application varied across studies. The most used protocols involved either a single intraoperative application (n=22; 33.33%) or a 7-day postoperative regimen (n=27; 40.9%). Overall, CHX was applied postoperatively in 42 studies (63.64%) and intraoperatively in 28 studies (42.42%).

The period and timing of CHX application also varied among studies; however, most of them established a 7-day application period (27 studies; 40.9%) or a single application on the day of the intervention (22 studies; 33.33%). On the other hand, most studies applied CHX postoperative (32 studies, 48.48%) or intraoperative (22 studies, 33.33%). Additionally, there are studies (n=10, 15.15%) that have mixed the timing protocols for CHX administration, including preoperative application followed by postoperative use, administration immediately before surgery and continued for seven days postoperatively, among others combinations.

Critical analysis and evidence synthesis

Essential information, including the author and publication date, sample characteristics, and study design of each included article, is compiled in Supplement 4 (http://www.medicina.oral.com/carpeta/suppl4_27864). It also summarizes the wound healing parameters evaluated, along with the primary objectives and key outcomes of the selected studies that investigated the clinical implications of CHX use in enhancing oral wound healing following surgical interventions.

Supplement 5 (http://www.medicina.oral.com/carpeta/suppl5_27864) presents a synthesis of current evidence on CHX application protocols across the included studies, highlighting the primary outcomes for each evaluated parameter. A concentration of 0.12% showed significant improvements in wound healing in 72.73% of studies, while 0.20% CHX showed significant effects in 81.39%. Regarding formulation, CHX gel demonstrated significant outcomes in 75% of studies, and CHX rinse in 80.49%. Application period also influenced results: Immediate post-surgical use led to significant findings in 72.72% of studies, whereas a 7-day application protocol was effective in 70.07%. In relation to timing of application, both postoperative and intraoperative were significantly associated with favourable wound healing (81.25% and 68.18% respectively). Better results were obtained postoperatively, probably linked to a longer application time (i.e., several days). As for the combination of different application times, despite the large variability, almost all obtained significant results for CHX (90.00%).

**Figure 2:**
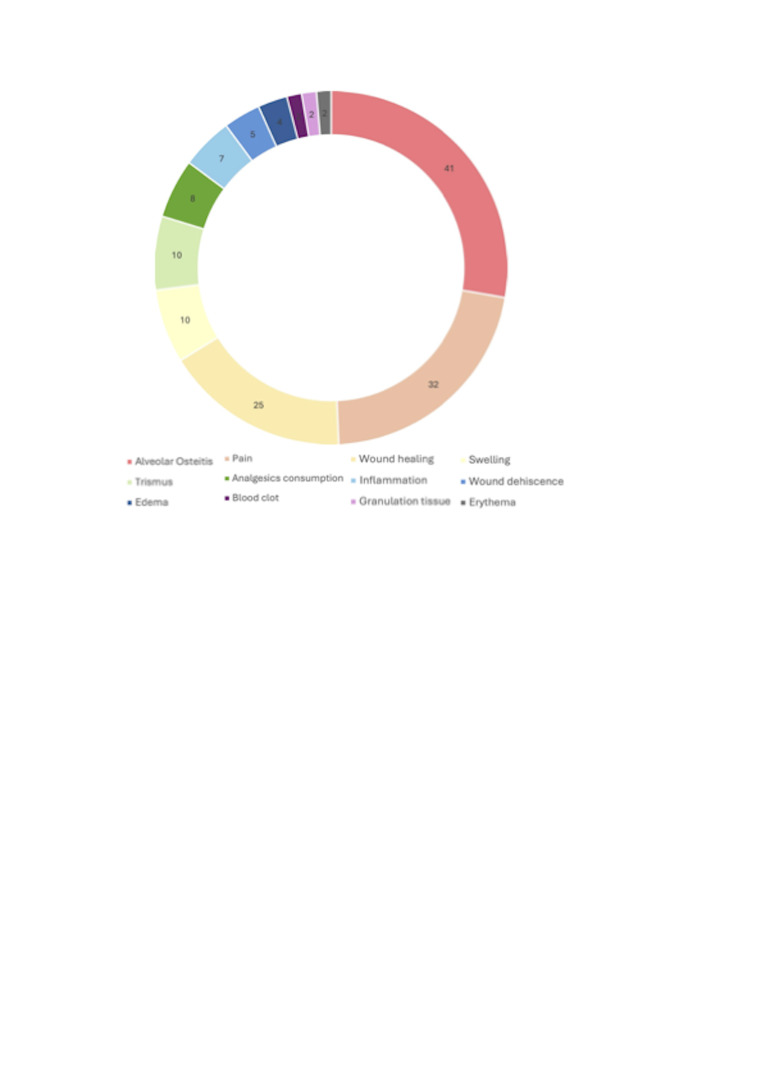
Pie chart graphically summarizing the available evidence on the outcomes investigated across primary-level studies. Outcomes were ordered by absolute counts and represented with colors for graphical purposes.

Regarding parameters related to oral wound healing after surgical procedures, Supplement 6 (http://www.medicina.oral.com/carpeta/suppl6_27864) compiles those assessed by the studies included in this scoping review: Alveolar osteitis, pain, wound dehiscence, wound healing, swelling, trismus, inflammation, edema, analgesic consumption, blood clot formation, granulation tissue, and erythema. The most frequently studied parameter was alveolar osteitis (n=41; 62.12%), with 73.17% of those studies reporting significant results in favor of CHX. Pain was the second most studied outcome (n=32; 48.48%), with 75% of studies showing significant effects. Wound healing followed (n=25; 37.87%), with 76% of those studies reporting significant improvements associated with CHX. The relative frequencies of all evaluated parameters are also graphically depicted in Figure 2.

2

Supplement 7 (http://www.medicina.oral.com/carpeta/suppl7_27864) presents the wound healing indices used by the studies included in this ScR, which represent a significant source of clinical and methodological heterogeneity. Currently, there is no universal consensus on which healing indices should be applied. Classical indices were identified, such as the Landry index (n=2), the Early Wound Healing Index by Wachtel et al. (n=3), the Madrazo-Jiménez index (n=2), and the Early Wound Healing Score by Marini et al. (n=1). The remaining studies employed non-standardized indices, which were classified as semi-quantitative scales.

## Discussion

The present scoping review, based on the analysis of 66 primary studies which included a total of 6,763 published cases, provides an updated synthesis of evidence on the efficacy of chlorhexidine (CHX) in different oral surgical contexts, allowing us to better understand its clinical implications and gaps in knowledge that may be useful in guiding future research. The control of infection in the postoperative period of oral surgical procedures remains one of the main challenges in clinical dentistry for ensuring proper wound healing. The persistence of microorganisms in the surgical site can inhibit or delay tissue repair by inducing sustained inflammation, impairing angiogenesis, and disrupting the early phases of collagen deposition and epithelial regeneration. This scenario highlights the importance of adjuvant therapeutic strategies aimed at optimizing healing and reducing complications, with CHX being the most widely studied antiseptic in this field.

Global efficacy of CHX in oral wound healing after oral surgery procedures

The results of this exploratory review reinforce the positive effect of CHX on wound healing in all types of oral surgical procedures investigated, i.e., third molar surgery (n=44 studies) and simple extractions (n=8 studies), periodontal surgery (n=10), dental implants (n=5 studies), oral biopsies (n=3 studies) and other fields of maxilofacial surgery (n=2 studies). Most available studies have been conducted in the context of third molar extraction (66.6% of included studies), a study model frequently chosen due to the high frequency of this intervention, standardized surgical technique, and the common occurrence of alveolar osteitis. The results of this scoping review are consistent with those of a meta-analysis previously published by our research group (13), in which CHX use was also significantly associated with improved wound healing outcomes (RR=0.66, 95% confidence intervals [CI]=0.55-0.80, p&lt;0.001). In that study the strongest evidence also came from trials conducted in the context of third molar surgery, reinforcing the robustness and clinical relevance of these findings. The overall consistency of favorable outcomes with CHX use suggests that its primary mechanism control of biofilm and reduction of bacterial load plays a decisive role in enhancing wound healing. By limiting microbial colonization, CHX helps to maintain an environment conducive to fibroblast proliferation, angiogenesis, and reepithelialization, ultimately accelerating the healing process. These findings align with the hypothesis that microbial imbalance is a key determinant of poor wound repair, and that antiseptic modulation during the critical early stages may shift the healing trajectory toward successful outcomes. Our findings indicate that the benefits observed in third molar extractions may extend to these other surgical scenarios, however, an existing evidence gap remains here, where future research is needed. This underscores the need for broader clinical trials addressing more diverse surgical settings, as wound healing is equally critical after oral biopsies, periodontal stability and successful oral implants survival (19).

CHX implications in the prevention of alveolar osteitis

Alveolar osteitis is the most frequently studied parameter according to this scoping review (n=41), reflecting its clinical relevance as one of the most painful and debilitating complications following tooth extraction. Results confirm that CHX significantly reduces the risk of alveolar osteitis, with more than 70% of studies reporting significant favorable results (p&lt;0.05). The results of our meta-analysis also demonstrate that the application of CHX significantly reduces the incidence of alveolar osteítis (13), with a 2.17-fold lower risk compared to controls (RR=0.46; 95%CI: 0.39-0.53; p&lt;0.001). On the other hand, previous meta-analytical studies also reported similar results (20 - 22), highlighting that our findings are consistent with higher-evidence studies. Alveolar osteitis development is related to different underlying factors, such as surgical trauma, patient-related risk factors (e.g., smoking, contraceptive use), and operator experience (23). The mechanisms underlying alveolar osteitis prevention by CHX likely involve both reduction of bacterial load and modulation of fibrinolytic activity. Several studies support the role of bacterial colonization in amplifying plasminogen activation and clot dissolution, leading to impaired healing (24). By reducing microbial proliferation, CHX may indirectly stabilize the clot and promote uneventful healing. Taken together, these observations strengthen the evidence and reinforce the routine recommendation of CHX use in clinical practice, especially after third molar surgery to prevent painful complications, where alveolar incidence is highest.

CHX and other clinical outcomes

Beyond overall wound healing and the prevention of alveolar osteítis, CHX has also been associated with improvements in other clinical parameters, singularly pain. Postoperative pain was consistently reduced after the application of CHX (n=32 studies), singularly after third molar surgery studies. It has been suggested that CHX use contributes to a more comfortable recovery period (25), although variability exists due to the subjective nature of pain assessment -commonly measured through subjective scales, such as visual analog scale (VAS)- and differences in follow-up schedules (13). Our scoping review also identified benefits in reducing swelling (n=10 studies), trismus (n=10 studies), analgesics consumption (n=8 studies), inflammation (n=7 studies), suture dehiscence (n=5 studies), edema (n=4 studies), granulation tissue (n=2 studies) and wound erythema (n=2 studies). Although evidence is weaker for these clinical parameters due to the smaller number of studies. This represents another important evidence gap that future research should address, since comprehensive evaluation of wound healing extends beyond alveolar osteitis and pain.

CHX formulation, concentration and administration protocols

An important issue in clinical practice relates to the pharmaceutical form of CHX. Our synthesis shows that both gel (n=29 studies) and rinse (n=36 studies) presentations have demonstrated significant positive results, with positive healing parameters for oral wounds in both scenarios (i.e., approximately 75% of gel-based studies and 80% of rinse-based studies reported improved outcomes). Our previous meta-analysis indicated that CHX gel was significantly more effective than mouth rinses (p&lt;0.001) (13). The enhanced performance of the gel formulation may be related to its longer persistence at the application site, which provides extended pharmacological activity compared with rinses (13). Nevertheless, this presentation has some practical limitations, since the actual contact time with the surgical wound cannot always be controlled, and in deeper or less accessible areas patients may encounter difficulties in achieving adequate application. Therefore, each pharmaceutical form may be more suitable for specific clinical situations (26). Gels offer localized delivery, higher bioavailability, and reduced adverse effects such as staining, making them ideal for procedures involving limited surgical fields such as single implant placement or localized extractions. In contrast, rinses are preferable when the surgical site is extensive, such as multiple extractions or wide periodontal flaps, given their ability to reach less accessible areas. This differentiation between vehicles reflects the importance of tailoring the antiseptic regimen to the clinical context rather than adopting a one-size-fits-all approach (26 , 27).

The effect of CHX is also concentration-dependent. Both 0.12% (n=21 studies) and 0.20% (n=41 studies) formulations have shown significant efficacy in this scoping review (i.e., respectively, 72.7% vs. 81.3% across primary-level studies with positive outcomes). Our previous meta-analysis also confirmed a significantly higher efficacy at a concentration of 0.20% vs 0.12% (p&lt;0.001) (13), which can be explained by its dose-dependent behavior, mainly exerting bacteriostatic activity at lower concentrations, while at higher concentrations it achieves a bactericidal effect (15). But their clinical indications could also differ, because lower concentrations have been proposed to be prescribed for extended periods (up to 4 weeks) to minimize adverse events (particularly mucosal irritation and tooth staining), and higher concentrations are suitable for short-term use (1-2 weeks), with higher rates (12).

Regarding administration protocols, singularly timing and duration of application, our scoping review indicates that postoperative use at home for 7 days is the most commonly studied and consistently effective protocol (n=27 studies). Intraalveolar application immediately after surgery (n=22 studies) has also been beneficial, with some studies suggesting that combining intraoperative placement with a postoperative regimen yields the most reliable results. In contrast, preoperative use alone (n=1 study) does not appear to provide significant benefits (28). However, these results have not been investigated in depth in meta-analyses due to the considerable heterogeneity in the clinical protocols used in primary studies (13). Therefore, CHX administration protocols also constitute an evidence gap that should be further investigated in the future in methodologically well-designed clinical trials with standardised methods.

CHX in combination with other agents

Another relevant aspect identified in our scoping review is the combination of CHX with bioactive agents aimed at enhancing wound healing beyond microbial control and better tissue repair. The most promising results have been reported for CHX combined with chitosan, although the number of available trials remains limited (n=4). When combined with CHX, the antiseptic effect of the latter complements the pro-healing environment induced by chitosan, resulting in superior clinical outcomes compared with CHX alone (p&lt;0.001). Our previous meta-analysis demonstrated the combination of CHX with chitosan resulted in a fourfold improvement in oral wound healing compared with controls, corresponding to a 75% reduction in the risk of complications. This effect was supported by a strong association (RR=0.25; 95% CI=0.17-0.37), and its efficacy was significantly superior to CHX alone (p&lt;0.001) (13). Chitosan has demonstrated properties that include stimulation of fibroblast proliferation, angiogenesis, and acceleration of epithelial closure (29 , 30 , 39 , 31 - 38). Despite the scarcity of evidence, the consistency and magnitude of effects reported justify further investigation of this synergistic approach.

Other associations have also been explored. The combination of CHX with hyaluronic acid has shown benefits in the late stages of healing by promoting fibroblast differentiation and extracellular matrix deposition, complementing the early antimicrobial effect of CHX (40). Trials have reported reduced pain, swelling, and improved tissue regeneration with CHX+ hyaluronic acid compared to CHX alone. Similarly, CHX combined with metronidazole has demonstrated potential in reducing alveolar osteitis incidence and postoperative pain, given the extended antibacterial spectrum achieved by this combination (24). Novel associations with platelet-rich fibrin (PRF), ADS systems to reduce staining, and boric acid solutions are emerging research lines that could provide alternative approaches with fewer side effects. However, the heterogeneity of results and the limited number of trials warrant cautious interpretation and further research.

This scoping review has potential limitations that should be adressed. First, sources of clinical and methodological heterogeneity limit the possibility of directly comparing results across studies and extract robust conclusions for stardardized protocols of administration. Second, the predominance of trials performed in the context of third molar surgery, with overrepresentation of this type of surgery in some way, restricts limits the generalizability of the findings to other oral surgical procedures. Third, the general effects of chlorhexidine on oral wound healing are well established in the literature and the novelty of the present manuscript is mainly limited to evidence synthesis and exploring gaps in knowledge across different surgical contexts and clinical protocols. Emerging associations were also discussed, particularly those involving synergistic combinations of chlorhexidine with bioactive agents such as chitosan and hyaluronic acid, and the standardized overview of concentration-dependent effects. In addition, the novelty of this review should be understood in relation to our previous meta-analysis on chlorhexidine in oral wound healing (13), which had a much narrower focus and more restrictive eligibility criteria, in line with meta-analytical studies. That earlier study was limited to randomized trials assessing wound healing improvement after oral surgical procedures (13). In contrast, the present scoping review adopts a broader approach, including studies that investigate combined therapies (e.g., with hyaluronic acid or antibiotics) and a wider range of outcomes not previously analyzed meta-analytically, such as swelling, trismus, or analgesic consumption. It also provides a more comprehensive examination of the various wound healing indices, for which no international consensus currently exists. Although this secondary-level study does not include statistical synthesis, as expected from its design, it benefits from the broader and more flexible framework of a scoping review, allowing a richer and more integrative mapping of the available evidence. On the other hand, a major strength of this review lies in its comprehensive approach, synthesizing evidence from a wide range of primary-level studies to provide an updated overview of CHX in oral wound healing. Additionally, the integration of findings with previous meta-analytical evidence reinforces the robustness and clinical applicability of the findings discussed here.

## Conclusions

This scoping review provides an updated overview of the clinical evidence regarding the use of CHX in oral surgical wound healing. The findings confirm that CHX contributes to improved wound healing outcomes and to the prevention of alveolar osteitis, while also showing benefits in reducing pain and other postoperative complications. Both gel and rinse formulations, as well as concentrations of 0.12% and 0.20%, have proven effective, although the choice should be tailored to the clinical context, balancing duration of use and potential adverse effects. Combinatory protocols, particularly intraoperative application followed by short-term postoperative use, appear promising, although further standardized studies are required. Moreover, the combination of CHX with other agents such as chitosan or hyaluronic acid has demonstrated enhanced efficacy, but the limited number of trials highlights the need for additional research. Taken together, these findings support the use of CHX as a practical, low-cost, and effective adjuvant in oral surgical procedures, while also identifying critical evidence gaps that should guide future well-designed clinical investigations.

## Figures and Tables

**Table 1:** Table Study sample characteristics of clinical trials included in this scoping review.

Total sample	66 studies6763 cases
Date of publication	
Range Min (year)Range Max (year)	19792024
Surgical approach*	
Third molars surgery	44
Periodontal surgery	10
Simple extraction	8
Implants	5
Biopsy	3
Maxilofacial surgery	2
Type of intervention*	
CHX	55
CHX + Others	10
CHX + Antibiotics	5
CHX + Hyaluronic Acid	5
CHX + Chitosan	4
CHX vehicle	
Rinse	36
Gel	29
Not reported	1
CHX concentration*	
0.20%	41
0.12%	21
1%	5
0.05%	1
0.5%	1
Application period	
7 days	27
Single application	22
15 days	9
>15 days	5
10 days	1
5 days	1
Not reported	1
Application timing*	
Postoperative	32
Intraoperative (intra-alveolar)	22
Mixed	10
Just before surgery	3
Preoperative	1

*More than one parameter was analysed per study.

## Data Availability

Declared none.
